# LEGMO: Extremity Salvage Using Extracorporeal Membrane Oxygenation

**DOI:** 10.1016/j.atssr.2022.11.002

**Published:** 2022-11-08

**Authors:** Nader Sarkis, Keith B. Allen, Brie McKiddy, Karthik Vamanan, Samantha Alsop

**Affiliations:** 1 Department of Cardiothoracic Surgery, Saint Luke's Mid America Heart Institute, Kansas City, Missouri; 2 Department of Vascular Surgery, Saint Luke's Mid America Heart Institute, Kansas City, Missouri

## Abstract

Mycotic aneurysm management balances the urgency of excising infected vasculature with the need to revascularize in or near an infected field. We present a case of a 47-year-old man with *Pseudomonas* sepsis, a failed kidney transplant, and a ruptured, previously stented right external iliac pseudoaneurysm. After excision of the infected pseudoaneurysm and stents, lower extremity revascularization was delayed through the innovative use of isolated limb perfusion using extracorporeal membrane oxygenation followed by staged extra-anatomic femoral-femoral bypass. This technique provided limb perfusion while allowing the patient’s sepsis to resolve to reduce the risk of recurrent infection after definitive revascularization.

Mycotic aneurysms and infected pseudoaneurysms require urgent intervention and can be complicated by rupture or systemic infection. Rupture mandates immediate excision of the affected vasculature with revascularization to maintain distal perfusion.^1^ In situ revascularization during active infection, however, significantly increases risk of postoperative graft infection, leaving extra-anatomic bypass grafting the modality of choice, especially in high-risk patients.^2^ We present a novel use of extracorporeal membrane oxygenation (ECMO) as a bridging therapy to maintain distal limb perfusion between excision of an infected pseudoaneurysm in the external iliac artery (EIA) and revascularization.

A 47-year-old man with a failed kidney transplant presented to our hospital with sepsis, a tender pulsatile right lower quadrant mass, and generalized weakness. The patient had a 3-year history of end-stage renal disease managed with peritoneal dialysis. Three months before presentation to our institution, the patient had undergone kidney transplantation complicated by right EIA dissection leading to an infarcted kidney transplant. Thrombectomy of the right EIA and explantation of the transplanted kidney were performed with need to place a bare-metal stent in the EIA to treat a localized dissection. The patient recovered and was discharged on aspirin and clopidogrel, and peritoneal dialysis was restarted. Four weeks later, the patient was rehospitalized for fevers and was found to have *Pseudomonas* bacteremia and an EIA pseudoaneurysm involving the previously placed bare-metal stent. The outside hospital implanted 3 iCAST (Atrium Medical) covered stents with effective isolation of the pseudoaneurysm, and the patient was discharged on antibiotics. Computed tomography performed 1 week later at the treating hospital demonstrated no evidence of pseudoaneurysm, and antibiotics were discontinued. The patient subsequently had recurrent fevers, malaise, and failure to thrive and presented to our institution with sepsis, a tender pulsatile right lower quadrant mass, healed right flank incision, and palpable pedal pulses.

Computed tomography angiography of the abdomen, pelvis, and lower extremities revealed a recurrent EIA pseudoaneurysm with a contained rupture (Figure A) with evidence for infection involving the previously placed iCAST and bare-metal stents; in-line arterial runoff to the right foot was noted. Blood culture was positive for gram-negative rods, with subsequent culture specimens growing *Pseudomonas*. Intravenous antibiotics were initiated, and plans for emergent operation to resect the infected ruptured pseudoaneurysm and previously placed vascular stents were undertaken. Considering the *Pseudomonas* sepsis and proximity of the infection to the inguinal ligament, limb salvage with in situ reconstruction or immediate femoral-femoral reconstruction was deemed to be high risk for recurrent infection. The plan was to provide isolated limb perfusion using ECMO with delayed revascularization once sepsis was resolved.

**Figure:**
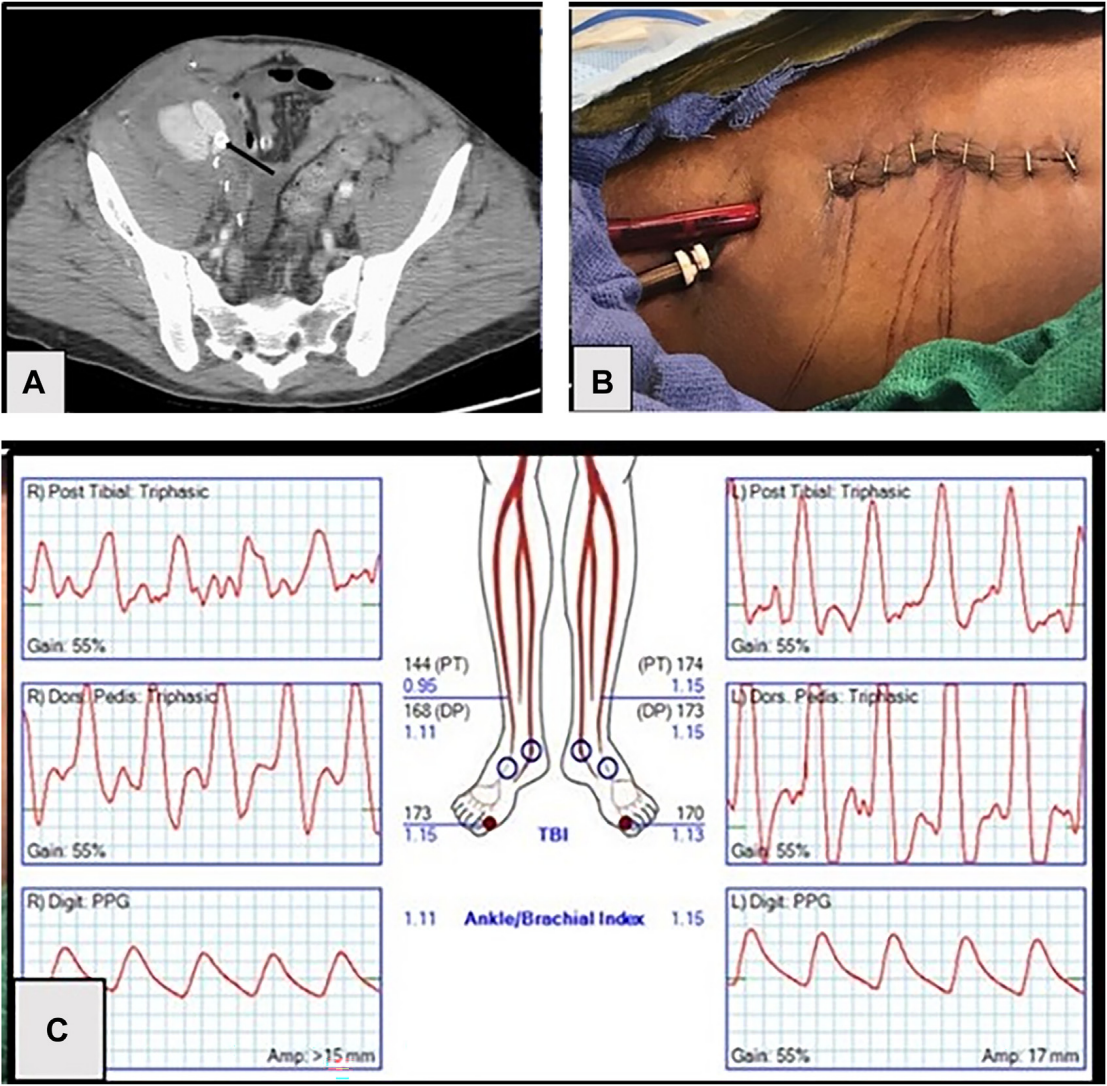
(A) Contained rupture of infected external iliac artery pseudoaneurysm with previously placed covered stents visible (arrow). (B) Extracorporeal membrane oxygenation cannulation of the superficial femoral artery and vein through a separate upper thigh incision. (C) Postoperative Doppler study demonstrating normal lower extremity waveforms and ankle/brachial index. (DP, dorsalis pedis; PPG, photoplethysmography; PT, posterior tibial; TBI, toe/brachial index.)

The right superficial femoral artery and vein were exposed through a longitudinal incision in the midthigh, avoiding the groin and future site for femoral-femoral bypass and to facilitate decannulation Percutaneous access was obtained through the skin distal to the incision with placement of 11F sheaths in the exposed vein and artery. The arterial access was then used to obtain vascular control of the contained rupture by placing a covered iliac stent that would ultimately be resected. An 0.035-inch wire was directed into the abdominal aorta and a 10 mm × 10 cm Viabahn (Gore) graft was positioned and deployed, providing vascular control and effectively excluding the pseudoaneurysm. No angiographic filling of the pseudoaneurysm was noted, and the right lower quadrant mass was no longer pulsatile. The retroperitoneum, which was exposed through the prior flank incision, revealed a grossly infected field with stents visible through the eroded iliac artery. Gross contamination and proximity to the groin were thought to preclude in-line reconstruction or immediate femoral-femoral reconstruction. After resection of all infected material, which required oversewing of the common, internal, and external iliac arteries, the wound was packed with antibiotic-soaked laparotomy pads.

Distal limb perfusion was now initiated using ECMO by exchanging the previously placed superficial femoral vein and artery sheaths, respectively, with a 12F cannula (Edwards) and a 17F × 30-cm-long multihole cannula (Medtronic) with the tip positioned in the inferior vena cava (Figure B). ECMO was initiated at 800 mL/min, and a nonpulsatile Doppler signal was noted in the right foot with stable hemodynamics; the thigh incision was closed after the cannula insertion sites were reinforced with purse-string sutures. Retroperitoneal packing was exchanged on postoperative day 3, and on postoperative day 5, after local control of infection and resolution of sepsis, the patient was decannulated and a femoral-femoral bypass constructed for definitive revascularization. The patient was discharged on postoperative day 10 with normal extremity perfusion (Figure C). Follow-up at 3 years demonstrated palpable pedal pulses without evidence of recurrent infection.

## Comment

Standard of care for mycotic aneurysms and pseudoaneurysms involves excision of the infected vascular segment with revascularization to maintain distal perfusion. Complications such as systemic infection and inopportune aneurysm location generally dictate the choice of operation. Several techniques exist for revascularization, including in-line reconstruction, endovascular repair, and extra-anatomic bypass grafting. In-line reconstruction has been demonstrated to have improved mortality, amputation, and patency compared with other bypass methods, whereas endovascular repair, if possible, may reduce postoperative pain and recovery time, sometimes serving as a bridge to more definitive repair. Both methods, however, involve graft placement within or near the infected field and are contraindicated in cases in which gross pyogenic infection is suspected.^3^

Whereas axillofemoral and femoral-femoral bypasses are commonly used for mycotic aneurysms in the aorta and iliac arteries, infrarenal mycotic aneurysms and infections in proximity to the groin are at higher risk for development of recurrent graft infections when performed immediately, particularly with gram-negative infections.^4^^,^^5^ *Pseudomonas* mycotic aneurysms, although relatively rare compared with other bacterial causes, are significantly less likely to resolve without complications, including higher mortality and lower limb salvage rates.^6^^,^^7^ The patient in this case had both a mycotic pseudoaneurysm in the EIA and blood cultures positive for *Pseudomonas*.

The innovative use of ECMO in this case allowed limb perfusion to be maintained while both local and systemic infection control could be achieved before revascularization with the goal of reducing recurrent graft infections. This case illustrates how a multidisciplinary approach involving vascular and cardiac surgeons, perfusion services, and critical care specialists working together can achieve superior results. Although the use of ECMO in this case was successful, a key limitation of generalization of this technique is the need for a hospital infrastructure that can support ECMO.
